# Irisin is a predictor of sarcopenic obesity in type 2 diabetes mellitus: A cross-sectional study

**DOI:** 10.1097/MD.0000000000026529

**Published:** 2021-07-02

**Authors:** Ayten Oguz, Murat Sahin, Dilek Tuzun, Ergul B. Kurutas, Cansu Ulgen, Ozlem Bozkus, Kamile Gul

**Affiliations:** a Faculty of Medicine, Istinye University, Istanbul; b Department of Endocrinology and Metabolism; c Department of Biochemistry; d Department of Internal Medicine, Faculty of Medicine, Kahramanmaras Sutcu Imam University, Kahramanmaras, Turkey.

**Keywords:** body composition, myokine, obesity, type 2 diabetes

## Abstract

We aimed to evaluate sarcopenia and sarcopenic obesity (SO) in patients with type 2 diabetes mellitus (T2DM), possible relationships with serum irisin and myostatin levels, and the effect of glycemic control on SO.

Ninety T2DM patients were included in this a cross-sectional study. Sarcopenia was determined by evaluating muscle mass (bioelectrical impedance analysis), muscle strength (HGS), and gait speed (GS). Patients with muscle mass loss with functionally reduced muscle strength and/or performance were considered sarcopenic. In addition, participants were divided into 3 groups according to the FM (fat mass)/FFM (fat-free mass) ratio [group 1:5th-50th percentiles; group 2:50th-95th percentiles and group 3: ≥95 percentiles (sarcopenic obese)]. Irisin, myostatin levels and metabolic parameters were measured in all patients.

The prevalence of sarcopenia and SO was 25.6% and 35.6%, respectively. Irisin levels were lower in sarcopenic patients, while glycosylated hemoglobin (A1c), body mass index (BMI), FM, and FM index were higher (*P* < .05). From group 1 to group 3, BMI, FM, FM index, GS, myostatin, and A1c increased, and muscle mass percentage, HGS, and irisin decreased (*P* < .05). A positive correlation was found between FM/FFM and myostatin and a negative correlation between FM/FFM and irisin (r = 0.303, *P = *.004 vs. r = −0.491, *P < *.001). Irisin remained an important predictor of SO, even after adjusting for confounding variables (OR:1.105; 95% CI:0.965–1.338, *P = *.002). The optimal cut-off value for irisin to predict SO was 9.49 ng/mL (specificity = 78.1%, sensitivity = 75.8%). In addition, A1c was an independent risk factor for SO development (OR:1.358, *P = *.055).

This study showed that low irisin levels (<9.49ng/mL) and poor glycemic control in T2DM patients were an independent risk factor, especially for SO.

## 1 Introduction

Body composition changes with aging, muscle mass and muscle strength decrease, and body fat percentage increases. Sarcopenia is defined as a generalized loss of muscle mass and strength that may cause health problems.^[1,2]^ Patients with type 2 diabetes mellitus (T2DM) have a higher risk.^[3,4]^ Recently, the definition of sarcopenic obesity (SO), which includes both sarcopenia and obesity, has also emerged. Sarcopenic obesity is a clinical and functional condition characterized by the association of sarcopenia, in which lean body mass decreases and fat mass (FM) increases, causing more serious metabolic disorders than obesity and sarcopenia alone. SO is also more common in patients with T2DM.^[5–7]^

Myostatin is a member of the transforming growth factor-β family, and although it is abundant in muscles, it is also found in small amounts in adipose tissue and heart muscle.^[8]^ Some studies revealed an association between increased myostatin levels and increased age, decreased muscle mass, and muscle strength.^[9,10]^ In addition, high myostatin levels have been shown to be associated with obesity and insulin resistance (IR).^[11,12]^

Irisin is a myokine that is released by skeletal muscles and is a potential biomarker for sarcopenia.^[13]^ Myostatin inhibition has been shown to increase irisin levels in animal studies.^[14]^ Furthermore, irisin has been associated with decreased body weight and increased insulin sensitivity.^[15]^ In sarcopenic individuals, circulating irisin levels have been shown to be lower and therefore recommended as a potential marker for sarcopenia.^[13,16]^

T2DM is associated with a rapid loss of skeletal muscle mass and strength.^[3,4]^ Some studies have shown an association between sarcopenia and diabetes duration and poor glycemic control.^[17,18]^ It has even been suggested that sarcopenia has the third complication in addition to the microvascular and macrovascular complications of diabetes.^[4,19]^ However, the causal relationship between SO and glycemic control remains unclear.

Sarcopenic obesity has different definitions. Diagnostic criteria and limits are not universally defined.^[20,21]^ However, several recent studies have suggested using the FM/fat-free mass (FFM) ratio for defining SO. FM/FFM is a more integrated index for the evaluation of abnormal body composition than indices of each individual component.^[22,23]^

Our aim was to evaluate the factors related to sarcopenia and SO in T2DM patients and to explore its association with serum levels of myostatin and irisin.

## 2 Material and methods

This cross-sectional study was conducted at the Endocrinology and Metabolism and Medical Biochemistry Clinics of Kahramanmaras Sutcu Imam University. The local ethics committee approved the observational cross-sectional study (dated: February 20, 2019; decision number:18), and written informed consent was obtained from all subjects.

### 2.1 Study protocol and inclusion criteria

Sample size calculations were performed using the G power analysis system.^[24]^ 93 T2DM patients (admitted to the endocrinology outpatient clinic) between the ages of 18–70 and a body mass index (BMI) of 25–40 kg/m^2^ were included in the study. Sociodemographic data, medications, comorbidities, chronic vascular complications, and diabetes self-management parameters (blood glucose measurements, dietary compliance, and exercise) of all patients were recorded.

**Figure 1:**
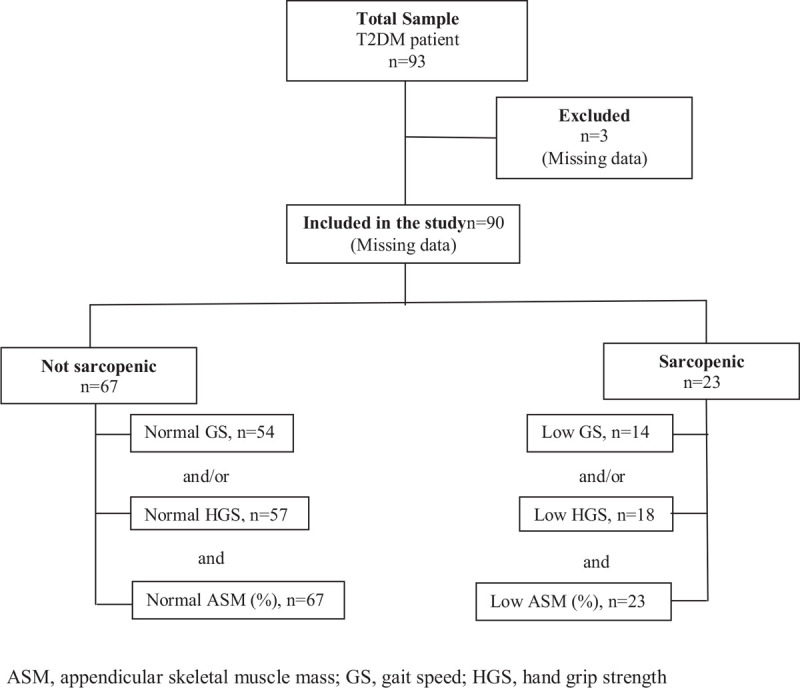
The selection of participants and evaluation for sarcopenia.

The patients included in the study were evaluated according to muscle mass and muscle strength and/or performance ^[1]^ (Fig. 1). In addition, irisin and myostatin levels and metabolic parameters were measured in all patients.

### 2.2 Exclusion criteria

Patients that use non-steroid anti-inflammatory drugs or prednisolone (>7.5 mg/day) and have contraindications for bio-impedance analysis (BIA) (e.g., pacemaker) were excluded from the study. Patients with type 1 diabetes, renal impairment (estimated glomerular filtration rate <15 ml/ min/1.73 m^2^), renal replacement therapy, pregnancy, infectious diseases, muscular dystrophy, lipodystrophy and cancer, Cushing syndrome, growth hormone, and severe vitamin D deficiency (<10 ng/ml), hypogonadism, hypothyroidism, and hyperthyroidism that could cause sarcopenia were also excluded.

### 2.3 Anthropometric measurements

During weight measurement, patients had light clothes and measurements were made with 0.1 a sensitivity. Height (m) was measured using a wall-mounted meter with a 0.1 cm sensitivity. We measured the waist circumference (WC) by locating the hip bone and the top of the right iliac crest, and then placing a measuring tape in a horizontal plane around the abdomen at the level of the iliac crest.^[25]^ Hip circumference (HC) (cm) was measured from the widest part of the hip. The waist-to-hip ratio (WHR) was calculated as the ratio of waist circumference to hip circumference. The bilateral maximum calf circumference (CC) and middle arm circumference (MAC) were also measured. MAC was measured at the midway point between the olecranon process of the ulna and the acromion process of the scapula. CC was measured as the maximum horizontal distance around the left calf as the subject stood upright.^[26]^

### 2.4 Body composition measurements

Body composition was measured using a BIA-device (TANITA DC 360 ST, Japan). Patients were asked not to eat, not to drink and not to undertake any physical activity at least three hours before the test and to void the bladder immediately before the measurement. FM (kg), FFM (kg), and appendicular skeletal muscle (ASM) (kg) were measured using BIA.

The skeletal muscle index (SMI) was calculated as ASM (kg)/height^2^ (kg/m^2^).^[1]^

ASM (%) was calculated as ASM (kg)/body kg × 100. The cut-off point for low ASM (%) was <37.0% for men and <27.6% for women.^[27]^

FFM index (FFMI), FFM/height^2^ (kg/m^2^), and FM index (FMI) were calculated as FM/height^2^ (kg/m^2^).^[1]^

### 2.5 Muscle strength evaluation

Muscle strength was evaluated using a hand-grip dynamometer (Kyto EH 101, Guangdong, China). While taking the measurement, the patients were asked to stand upright, and they were asked to grasp and tighten the tool with all their strengths. The measurement was performed three times from both hands (right and left), and the mean of these values was recorded.^[28]^ The hand grip strength (HGS) measurement results were taken as kg and the device is sensitive to 0.1 kg and can make measurements between 5–90 kg.

Cut-off values for low mass strength according to HGS were adjusted to BMI were as follows: ≤29 kg, ≤30 kg, ≤30 kg, and ≤32 kg (according to these BMI groups, ≤24, 24.1–26, 26.1–28, and > 28 kg/m^2^, respectively) for males and ≤17 kg, ≤17,3 kg, ≤18 kg, and ≤21 kg (according to these BMI groups, 23, 23.1–26, 26.1–29, and >29 kg/m^2^, respectively) for females.^[29]^

Poor physical performance was evaluated using a gait speed (GS) test (6 m). In this test, the participants were requested to walk at their own pace in a pre-measured area of 6 m. The duration was started with the person's foot at the start line and ended when it crossed the finish line. Two measurements were performed, and the best result was recorded in seconds (s).^[30]^ The GS scores were adjusted for height. Cutoff values for poor physical performance were GS≥7 s (height≤173 cm) and GS≥6 s (height>173 cm) for males and GS≥7 s (height≤159 cm) and GS≥6 s (height>159 cm) for females.^[29]^

### 2.6 Definition of sarcopenia and sarcopenic obesity:

Participants were identified as being “not sarcopenic” or “sarcopenic” using the criteria recommended by the European Working Group on Sarcopenia in Older People.^[1]^ Patients with muscle mass loss with functionally reduced muscle strength and/or performance were considered sarcopenic. As the patients included in the study were overweight/obese, we used ASM (%) as the muscle mass while defining sarcopenia.^[7]^

The FM/FFM ratio is an index of the SO. Participants were divided into 3 groups according to the FM/FFM ratio and used the following cut-off values (5th-95th percentiles) from National Health and Nutrition Examination Survey III obtained using BIA:^[31]^ Participants were divided into 3 groups according to FM/FFM values (adjusted to gender, BMI and age).

Group 1 (control); cut-off values for FM/FFM (5th-50th percentiles)were <0.34, <0.43; <0.34, <0.42; <0.36, <0.43 (according to BMI 25–29.9 and 30–39.9 kg/m^2^, respectively) for 18–40, 40–60 and 60–70 years males and <0.59, <0.76; <0.62, <0.77; <0.63, <0.76 (according to BMI 25–29.9 and 30–39.9 kg/m^2^,respectively) for 18–40, 40–60 and 60–70 years females.

Group 2; increase in FM are small relative to those in FFM. Cut-off values for FM/FFM (50th-95th percentiles) were 0.34–0.46, 0.43–0.57; 0.34–0.47, 0.42–0.59; 0.36–0.50, 0.43–0.59 (according to BMI 25–29.9 and 30–39.9 kg/m^2^, respectively) for 18–40, 40–60 and 60–70 years males and 0.59–0.74, 0.76–1.01; 0.62–0.76, 0.77–0.93; 0.63–0.76, 0.76–0.97 (according to BMI 25–29.9 and 30–39.9 kg/m^2^, respectively) for 18–40, 40–60 and 60–70 years females.

Group 3; SO phenotypes, where FM is greatly increased and FFM is decreasing. Cut-off values for FM/FFM (≥95th percentiles) were >0.46, >0.57; >0.47, >0.59; <0.50, >0.59 (according to BMI 25–29.9 and 30–39.9 kg/m^2^, respectively) for 18–40, 40–60 and 60–70 years males and >0.74, >1.01; >0.76, >0.93; >0.76, >0.97 (according to BMI 25–29.9 and 30–39.9 kg/m^2^,respectively) for 18–40, 40–60 and 60–70 years females.

### 2.7 Biochemical measurements

Blood samples for biochemical parameters were taken from the antecubital vein between and 08:00–09:00 in the morning after 8–10 h of fasting. Glucose, alanine aminotransferase (ALT), creatinine (Cr), and lipid parameters were measured by spectrophotometry using the Advia 1800 Chemistry System (Siemens, Germany). Glycosylated hemoglobin (A1c) was measured usinga high-pressure liquid chromatography (HPLC) device and a commercial kit (BioRad D-10 Hemoglobin Testing System, France). Spot urine Cr levels were measured based on the reaction of Cr with picric acid using a biochemistry analyzer and commercial kit (ADVIA Chemistry Cr Concentrated CRE_2c). Urine protein/Cr levels were expressed as mg/gr.

Myostatin and irisin levels in the samples were measured in duplicate using commercially available solid-phase sandwich enzyme-linked immunosorbent assay (ELISA) kits (Myostatin and Irisin kits were obtained from MyBioSource Company, USA) according to the manufacturer's protocol. Human myostatin ELISA kit (Cat. No: MBS021687) sensitivity was 1.0 ng/mL. Both intra-assay CV (%) and inter-assay coefficients of variability (CV) (%) variabilities were less than 15%. The Human Irisin ELISA Kit (Cat. No: MBS706887) sensitivity was 0.78 ng/mL. Intra-assay CV (%) and inter-assay CV (%) were less than 8% and 10%, respectively.

Normal reference values were as follow; total cholesterol (Total-C) 0–200 mg/dl, triglyceride (TG) 0–150 mg/dl, high density lipoprotein (HDL-C) 26–86 mg/dl, low density lipoprotein (LDL-C) 0–130 mg/dl, ALT 7–45 U/L, Cr 0.5–0.9 mg/dl, and spot urine protein/Cr 50–200 mg/gr, irisin 3.12–200 ng/ml and myostatin 3.12–100 ng/ml.

### 2.8 Statistical analysis

Data are presented as the mean±standard deviation (SD), unless otherwise specified. Data were analyzed using IBM SPSS (Statistical Package for Social Sciences) version 25. The Kolmogorov–Smirnov test was used to determine whether the samples had a normal distribution and whether the variances were homogeneous. An independent 2 Sample *t*-test was used to compare the two groups in the data with normal distribution, and the Mann–Whitney *U* test, a nonparametric test, was used in the data without normal distribution. A one-way ANOVA test was used to evaluate more than two groups. Data that differed between the groups were evaluated using post-hoc analysis. If the variance analysis was homogeneous, the group number was three, but the sample was not equal, and the Scheffe method was used to evaluate the difference in significance in the post-hoc analysis. The direct relationship between the variations was evaluated using Pearson's and Spearman's correlation tests. The chi-square test was used to evaluate the association between the frequency distribution of categorical variables. Logistic regression analysis was performed to determine the effects of clinical and laboratory variables on SO. The variables found to be statistically significant in the univariate analysis and other potential confounders were used in the multiple logistic regression model with the enter method to determine the independent prognostic factors of SO. Relative odds were expressed as odds ratios (ORs) and confidence intervals (CIs). Statistical significance was set at *P* < .05. The values of the predicted irisin levels and 95% CIs were computed. After performing the antilogarithmic transformation, the cutoff values and 95% CIs were determined.

## 3 Results

Initially, 93 patients were included in the study, but three patients were excluded because of missing data. This study included 90 patients, 22.2% (n = 20) male and 77.8% (n = 70) female (Fig. 1).

### 3.1 Sarcopenia evaluation

When the patients included in the study were evaluated according to muscle mass and muscle strength and/or performance, the rate of sarcopenia was 25.6% (n = 23). When analyzed by sex, 15.0% (n = 3) of men and 28.6% (n = 20) of women were sarcopenic, but the difference was statistically insignificant (*P = *.201). The percentage of patients with low muscle mass for men and women (15.0% vs 11.4%) and poor physical performance (high GS (s) for men and women, 30.0% and 54.0%, respectively) between males and females were insignificant (*P = *.460 vs *P = *.086). The percentage of patients with low HGS (kg) was significantly higher in men than in women (54.3% vs 25.0%, respectively, *P = *.018).

There was no statistically significant difference between sarcopenic and non-sarcopenic type 2 diabetic patients in terms of baseline characteristics (Table 1).

**Table 1:** Baseline characteristics of T2DM patients with and without sarcopenia.

Parameters	Non-sarcopenic (n = 67, 74.4%)	Sarcopenic n = 23, 25.6%)	*P*
Age (years)	55.01±8.81	54.17 ± 7.68	.685
Gender n, (%)			
Female	50 (71.4)	20 (28.6)	.201
Male	20 (85.0)	3 (15.0)	
DM duration (years)	10.97 ± 6.74	11.26 ± 6.03	.848
Smoking, n (%)	4 (6.0)	1 (4.3)	.909
Education level, n (%)			
≤ Elementary school	50 (74.6)	20 (87.0)	.201
≥Elementary school	17 (25.4)	3 (13.0)	
Medication, n (%)			
OAD	26 (38.8)	7 (30.4)	.468
Insulin	41 (61.2)	16 (69.6)	.617
Statin	43 (64.2)	16 (69.6)	.962
MIVC, n (%)			
Neuropathy	36 (53.7)	13 (56.5)	.816
Retinopathy	17 (25.4)	9 (39.1)	.217
Nephropathy	3.0 (4.5)	1.0 (4.3)	.731
MAVC, n (%)			
ASCVD	19 (28.4)	8 (34.8)	.189
PAD	1 (1.5)	1 (4.3)	.633
CVD	1 (1.5)	1 (4.3)	.633
Comorbidities, n (%)			
Hypertension	40 (59.7)	14 (60.9)	.921
Hyperlipidemia	46 (68.7)	18 (78.3)	.371
Obesity	52 (77.6)	21 (91.3)	.125
Diabetes self-management, n (%)			
SMBG	39 (58.2)	14 (60.9)	.823
Diet compliance	32 (47.8)	11 (47.8)	.996
Exercises	23 (34.3)	7 (30.4)	.802

Data are presented as n (%) and mean ± standard deviation. T2DM = Type 2 Diabetes Mellitus; OAD = oral antidiabetics; MIVC = microvascular complications; MAVC = macrovascular complications; ASCVD = atherosclerotic cardiovascular disease; PAD = peripheral arterial disease; CVD = cerebrovascular disease; SMBG = Self Management Blood Glucose Monitoring.

When sarcopenic and non-sarcopenic T2DM patients were compared in terms of anthropometric, body composition, and laboratory parameters (Table 2), mean BMI, WC, HC, FM, FMI, and CC were higher in sarcopenic patients (*P = *.003, *P = *.025, *P = *.013, *P = *.017, *P = *.002, and *P = *.001), but ASM (%), (*P = *.002), and HGS (*P = *.009) were lower in sarcopenic patients. A1c levels were higher in sarcopenic patients, and the rate of patients above target A1c (≥7%) was significantly higher in the sarcopenic group (*P = *.016 vs *P = *.002).

**Table 2:** Anthropometric, body composition and laboratory parameters of T2DM patients with and without sarcopenia.

Parameters	Non-sarcopenic (n = 67, 74.4%)	Sarcopenic (n = 23, 25.6%)	*P*
BMI (kg/m^2^)^∗^	32.46 ± 5.80	36.85 ± 5.93	**.003**
WC (cm)^∗^	110.69 ± 11.96	117.49 ± 12.37	**.025**
HC (cm)^∗^	113.17 ± 12.70	121.05 ± 12.11	**.013**
WHR (cm)^∗^	0.97 ± 0.082	0.97 ± 0.084	.623
CC (cm)^∗^	40.92 ± 5.51	44.13 ± 5.31	**.017**
MAC (cm)^∗^	35.62 ± 4.30	37.26 ± 5.15	.139
FM (kg)^∗^	32.52 ± 12.21	42.43 ± 12.76	**.002**
FFM (kg)^∗^	51.18 ± 7.96	50.80 ± 7.72	.843
ASM (kg)^∗^	28.97 ± 4.50	28.75 ± 4.37	.842
ASM (%)^∗^	35.14 ± 5.32	31.28 ± 4.08	**.002**
SMI (kg/m^2^)^∗^	11.17 ± 1.22	11.33 ± 0.96	.587
FMI (kg/m^2^)^∗^	12.75 ± 5.06	16.83 ± 4.97	**.001**
FFMI (kg/m^2^)^∗^	19.74 ± 2.16	20.02 ± 1.70	.586
GS (s)^∗^	6.62 ± 1.80	6.74 ± 1.61	.776
HGS (kg)^∗^	25.11 ± 8.96	19.65 ± 6.39	**.009**
Myostatin^∗^	44.95 ± 13.47	48.94 ± 8.68	.108
Irisin^∗^	13.67 ± 9.07	10.07 ± 6.17	**.038**
FPG (mg/dl)^∗^	164.82 ± 60.23	180.69 ± 56.33	.259
A1c (%)^∗^	7.99 ± 1.81	9.09 ± 1.96	**.016**
< 7 (n,%)^†^	28 (41.8)	2 (8.7)	**.002**
≥7 (n,%)^†^	39 (58.2)	21 (91.3)	
Total-C (mg/dl)^∗^	171.56 ± 40.38	186.69 ± 45.82	.169
LDL-C (mg/dl)^∗^	111.89 ± 38.82	129.52 ± 36.13	.054
HDL-C (mg/dl)^∗^	44.79 ± 10.34	42.76 ± 10.08	.398
TG (mg/dl)^‡^	167.82 ± 91.00	170.65 ± 82.91	.937
ALT (U/L)^∗^	23.02 ± 11.17	25.78 ± 17.16	.478
Cr (mg/dl)^‡^	0.74 ± 0.18	0.67 ± 0.16	.279
eGFR (ml/min)^‡^	109.78 ± 37.98	205.46 ± 79.10	.523
Spot urine protein/Cr (g/g)^‡^	94.60 ± 17.10	98.14 ± 11.34	.627

Continuous variables were expressed as the mean ± SD; categorical variables were expressed as a number (percentage).

∗ Independent samples *t*-tests.

† Chi-square χ^2^ test.

‡ Mann–Whitney *U* test. *P* < .05 is significant. BMI = body mass index; WC = waist circumference; HC = hip circumference; WHR = waist–hip ratio; CC = calf circumference; MAC = mid-arm circumference; FM = fat mass; FFM = fat-free mass; ASM = appendicular skeletal muscle mass; SMI = skeletal muscle index; FMI = fat mass index; FFMI = fat-free mass index; GS = gait speed; HGS = hand grip strength; FPG = fasting plasma glucose; A1C = glycosylated hemoglobin; Total-C = total cholesterol; HDL-C = high-density lipoprotein cholesterol; TG = triglyceride; ALT = alanine aminotransferase; Cr = creatinine; eGFR = glomerular filtration ratio.

### 3.2 Evaluation of sarcopenic obesity

**Figure 2:**
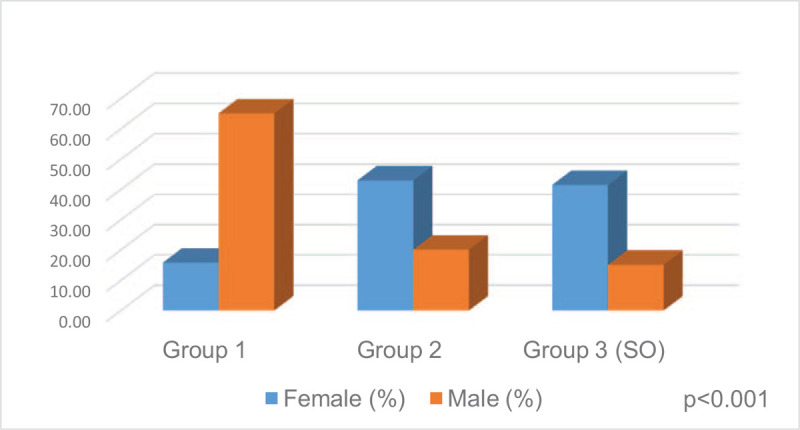
Sex distribution of groups according to FM/FFM.

The patients included in the study were divided into three groups: group 1 (n = 24, 26.7%), group 2 (n = 34, 37.8%), and group 3 (SO) (n = 32, 35.6%) according to the FM/FFM rate (Table 3). In terms of gender, the male ratio was higher in group 1, but the female ratio was higher in groups 2 and 3 (*P* < .001) (Fig. 2). From group 1 to group 3, BMI, WC, HC, CC, MAC, FM, FM (%), FM/FFM, FMI, GS, A1c, and total-C increased (¶*P < *.001, *P < *.001, *P < *.001, *P < *.001, *P < *.001, *P < *.001, *P < *.001, *P < *.001, *P < *.001, *P < *.003, *P = *.009 and *P = *.032, respectively), but ASM (kg), ASM (%), FFM (%), FFMI and HGS were decreased, and the differences were statistically significant (¶*P = *.004, *P < *.001, *P* < .001, *P = *0.019 and *P* < .001, respectively).

**Table 3:** The anthropometric, body composition and laboratory characteristics of the patient by fat to fat-free mass ratio (adjusted age, sex and BMI).

Parameters	Group 1 n = 24, 26.7%	Group 2 n = 34, 37.8%	Group 3 n = 32, 35.6%	*P*^¶^
Gender^∗^				<**.001**
Female	11 (15.7)	30 (42.9)	29 (41.4)	
Male	13 (65.0)	4 (20.0)	3 (15.0)	
Age (year)^†^	54.62 ± 8.74	54.29 ± 9.41	55.46 ± 7.47	.852
BMI (kg/m^2^)^†^	27.37 ± 3.50	32.66 ± 3.78^||^	39.22 ± 4.39^§^^,^^||^	<**.001**
WC (cm)^†^	107.93 ± 9.04	116.32 ± 12.9^||^	125.37 ± 8.79^§^^,^^||^	<**.001**
HC (cm)^†^	110.3 ± 9.19	120.05 ± 11.92^||^	129.25 ± 9.35^§^^,^^||^	<**.001**
WHR (cm)^†^	0.97 ± 0.09	0.96 ± 0.07	0.97 ± 0.08	.889
CC (cm)^†^	37.79 ± 4.17	40.32 ± 3.84	46.21 ± 5.17^§^^,^^||^	<**.001**
MAC (cm)^†^	33.00 ± 4.12	34.97 ± 2.79	39.46 ± 4.28^§^^,^^||^	<**.001**
FM (kg)^†^	20.41 ± 4.41	32.51 ± 6.32^||^	48.74 ± 7.99^§^^,^^||^	<**.001**
FM (%)^†^	27.45 ± 4.84	39.85 ± 4.45^||^	48.59 ± 3.88^§^^,^^||^	<**.001**
FFM (kg)^†^	53.84 ± 9.30	48.88 ± 6.84	51.35 ± 7.22	.058
FFM (%)^†^	72.55 ± 4.82	60.15 ± 4.44^||^	51.40 ± 3.87^§^^,^^||^	<**.001**
FM/FFM^†^	0.38 ± 0.09	0.67 ± 0.11^||^	0.95 ± 0.14^§^^,^^||^	<**.001**
ASM (kg)^†^	30.48 ± 5.25	27.66 ± 3.87^||^	26.98 ± 2.66^||^	.004
ASM (%)^†^	41.06 ± 2.72	34.04 ± 2.51^||^	29.09 ± 2.19^§^^,^^||^	<**.001**
SMI (kg/m^2^)^†^	11.22 ± 1.46	11.08 ± 1.04	10.43 ± 1.65	.074
FMI (kg/m^2^)^†^	7.58 ± 1.85	13.14 ± 2.75^||^	19.15 ± 3.29^§^^,^^||^	<**.001**
FFMI (kg/m^2^)^†^	19.82 ± 2.59	19.58 ± 1.83	18.13 ± 2.88^||^	**.019**
GS (s)^†^	6.20 ± 1.55	6.41 ± 1.65	7.58 ± 1.66^§^^,^^||^	**.003**
HGS (kg)^†^	29.18 ± 10.86	22.50 ± 6.36^||^	20.87 ± 5.76^||^	<**.001**
Myostatin^†^	40.05 ± 15.91	47.55 ± 7.23	48.66 ± 12.68^||^	**.022**
Irisin^†^	17.54 ± 9.35	14.15 ± 8.13^||^	7.66 ± 5.27^§^^,^^||^	<**.001**
FPG (mg/dl)^†^	167.12 ± 61.70	169.20 ± 57.59	169.84 ± 61.28	.985
A1c (%)^†^	7.45 ± 1.06	8.17 ± 1.76	9.00 ± 2.28^||^	**.009**
Total-C (mg/dl)^†^	157.9 ± 35.17	177.20 ± 40.65	187.18 ± 44.49^||^	**.032**
LDL-C (mg/dl)^†^	108.83 ± 43.53	113.91 ± 35.05	124.71 ± 38.34	.285
HDL-C (mg/dl)^†^	47.32 ± 2.11	47.00 ± 2.25	42.86 ± 4.25	.494
TG (mg/dl)^‡^	154.54 ± 15.92	162.94 ± 13.69	185.00 ± 18.33	.403
ALT (U/L)^†^	21.66 ± 8.78	23.08 ± 12.83	25.96 ± 15.42	.442
Cr (mg/dl)^‡^	0.72 ± 0.15	0.71 ± 0.16	0.73 ± 0.22	.931
eGFR (ml/min)^‡^	100.83 ± 10.87	94.73 ± 17.37	92.33 ± 16.70	.130
Spot urine protein/Cr(g/g)^‡^	57.65 ± 9.92	74.72 ± 37.43	181.50 ± 14.45	.075

Group 1, control group, Group 2, increase in FM are small relative to those in FFM; Group 3, sarcopenic obese. Continuous variables were expressed as the mean ± SD; categorical variables were expressed as a number (percentage).

∗ Chi-square χ^2^ test.

† One-Way ANOVA test.

‡ Kruskal–Wallis H test. *P* < .05 is significant.

§ *P* < .05 vs group 2 and 3 by Scheffe's test.

|| *P* < .05 vs group 1 by Scheffe's test.

¶ *P* value for difference among the three groups in means (ANOVA). A1C = glycosylated hemoglobin, ALT = alanine aminotransferase, ASM = appendicular skeletal muscle mass, BMI = body mass index, CC = calf circumference, Cr = creatinine, eGFR = glomerular filtration ratio, F = female, FFM = fat-free mass, FFMI = fat-free mass index, FM = fat mass, FMI = fat mass index, FPG = fasting plasma glucose, GS = gait speed, HC = hip circumference, HDL-C = high-density lipoprotein cholesterol, HGS = hand grip strength, M = male, MAC = mid-arm circumference, SMI = skeletal muscle index, TG = triglyceride, Total-C = total cholesterol, WC = waist circumference, WHR = waist–hip ratio.

In post hoc analysis, BMI, WC, HC, FM (kg), FM (%), FM/FFM, FMI were higher (*P* < .001, *P = *.008, *P = *.005, *P* < .001, *P* < .001, *P* < .001, *P* < .001 vs *P* < .001, *P* < .001, *P* < .001, *P* < .001, *P* < .001, *P* < .001, *P* < .001), but FFM (%), ASM (kg), ASM (%), and HGS were lower in groups 2 and 3 than in group 1 (*P* < .001, *P = *.032, *P* < .001, *P = *.006 vs *P* < .001, *P = *.006, *P* < .001, *P = *.001). In addition, CC, MAC, GS, total-C and A1c levels were significantly higher in group 3 than in group 1 (*P* < .001, *P* < .001, *P = *.010, *P = *.033, and *P = *.010, respectively). BMI, WC, HC, CC, MAC, FM (kg), FM (%), FM/FFM and GS were significantly higher (*P* < .001, *P = *.008, *P* < .001, *P* < .001, *P* < .001, *P* < .001, and *P = *.018, respectively), and FFM (%) and ASM (%) was significantly lower in group 3 than in group 2 (*P* < .001 vs *P* < .001).

The relationship between demographic, anthropometric, and metabolic parameters and sarcopenia and SO markers in T2DM patients is shown in Table 4. There was a positive correlation between FM/FFM and FMI, GS, BMI, CC, MAC, Total-C, and A1c levels (r = 0.966, *P* < .001, r = 0.301, *P* < .001, r = 0.841, *P* < .001, r = 0.589, *P* < .001, r = 0.589, *P* < .001, r = 0.250, *P = *.017 and r = 0.250, *P = *.015, respectively), and a negative correlation between FM/FFM with ASM (%), FFMI, and HGS (r = −0.980, *P* < .001, r = −0.310, *P = *.003 and r = −0.385, *P* < .001). A positive correlation was detected between ASM (%) and FFMI, HGS (r = 0.311, *P* < .001 vs r = 0.426, *P* < .001), and a negative correlation was detected between ASM (%) and FMI, FM/FFM, GS, BMI, CC, MAC, total-C, and A1c (r = −0.947, *P* < .001, r = −0.988, *P* < .001, r = −0.296, *P = *.005, r = −0.815, *P* < .001, r = −0.564, *P* < .001, r = −0.552, *P* < .001, r = −0.281, *P = *.007 and r = −0.279, *P = *.008, respectively). There was a positive correlation between HGS and ASM (%), FFMI (r = 0.426, *P* < .001 vs r = 0.273, *P = *.009), negative correlation between HGS and FM/FFM, FMI, GS, BMI, total C, and A1c (r = −0.385, *P* < .001, r = −0.461, *P* < .001, r = −0.320, *P = *.002, r = −0.208, *P = *.049, r = −0.226, *P = *.032, and r = −0.259, *P = *.014, respectively). There was a positive correlation between GS and FM/FFM, FFMI, age, and A1c (r = 0.301, *P = *.004, r = 0.323, *P = *.002, r = 0.290, *P = *.006 and r = 0.240, *P = *.023, respectively), and a negative correlation between GS, ASM (%), FMI, and HGS (r = −0.296, *P = *.005, r = −0.209, *P = *.048, and. r = −0.320, *P = *.002, respectively).

**Table 4.** The relationship between demographic, anthropometric and metabolic parameters with sarcopenia and SO markers in T2DM.

Variables		FM/FFM^∗^	ASM (%)^∗^	FMI^∗^	FFMI^∗^	HGS^∗^	GS^∗^	Age^∗^	BMI^∗^	WHR^∗^	CC^∗^	MAC^∗^	FPG^∗^	T-C^∗^	LDL-C^∗^	HDL-C^∗^	TG^†^	Cr^†^	eGFR^†^	Myostatin^∗^	Irisin^∗^	A1c^∗^
FM/FFM	r	–	−0.988	0.966	−0.310	0.385	0.301	−0.010	0.841	−0.068	0.589	0.589	−0.003	0.250	0.113	0.203	0.039	−0.099	−0.148	0.303	−0.491	0.256
	*P*	–	**.000**	**.000**	**.003**	**.000**	**.004**	.928	**.000**	.523	**.000**	**.000**	.980	**.017**	.287	.055	.718	.355	.165	**.004**	**.000**	**.015**
ASM(%)	r	−0.988	–	−0.947	0.311	0.426	−0.296	0.027	−0.815	0.079	−0.564	−0.552	−0.024	−0.281	−0.119	0.221	−0.046	0.122	0.143	−0.351	0.494	−0.279
	*P*	**.000**	–	**.000**	**.003**	**.000**	**.005**	.801	**.000**	.459	**.000**	**.000**	.826	**.007**	.265	.056	.664	.252	.179	**.001**	**.000**	**.008**
HGS(kg)	r	−0.385	0.426	−0.461	0.273	–	−0.320	−0.081	−0.208	0.024	0.041	0.049	−0.051	−0.226	−0.184	0.181	−0.010	0.259	0.045	−0.270	0.354	−0.259
	*P*	**.000**	**.000**	**.000**	**.009**	–	**.002**	.447	**.049**	.825	.701	.649	.631	**.032**	.082	.087	.925	.054	.676	**.010**	**.001**	**.014**
GS(m/s)	r	0.301	−0.296	−0.209	0.323	−0.320	–	0.290	0.163	0.128	0.111	0.133	0.004	0.094	0.137	0.102	0.006	0.206	−0.378	0.070	−0.275	0.240
	*P*	**.004**	**.005**	**.048**	**.002**	**.002**	–	**.006**	.125	.229	.299	.211	.970	.379	.199	.340	.954	.051	.055	.513	**.009**	**.023**
FMI	*r*	0.966	−0.947	–	−0.169	−0.461	−0.209	−0.090	0.945	−0.160	0.693	0.689	0.017	0.232	0.127	0.180	0.090	−0.154	−0.089	0.121	−0.428	−0.185
	*P*	**.000**	**.000**	–	.111	**.000**	**.048**	.398	**.000**	.132	.000	.000	.874	.028	.231	.090	.400	.147	.407	.257	**.000**	.081
FFMI	*r*	−0.310	0.311	−0.169	–	0.273	0.323	−0.138	0.128	−0.023	0.112	0.081	−0.083	−0.036	0.180	0.188	0.173	0.224	0.012	−0.326	0.176	0.265
	*P*	**.003**	**.003**	.111	–	**.009**	**.002**	.196	.228	.829	.292	.449	.437	.734	.090	.066	.103	.034	.914	**.002**	.098	**.012**

∗ Pearson correlation test.

† Spearman correlation test. *P* < .05 is significant. A1C = glycosylated hemoglobin, ASM = appendicular skeletal muscle mass, BMI = body mass index, CC = calf circumference, Cr = creatine, eGFR = glomerular filtration ratio, FFMI = fat-free mass index, FM/FFM = fat mass/fat-free mass, FMI = fat mass index, FPG = fasting plasma glucose, GS = gait speed, HDL = high-density lipoprotein cholesterol, HGS = hand grip strength, LDL-C = low-density lipoprotein cholesterol, MAC = mid-arm circumference, SMI = skeletal muscle index, SO = sarcopenic obesity, T2DM = type 2 diabetes mellitus, T-C = total cholesterol, TG = triglyceride, WHR = waist–hip ratio.

### 3.3 Evaluation of myostatin and irisin

Myostatin levels were similar between patients with and without sarcopenia (*P = *.108). Irisin levels were significantly lower in patients with sarcopenia than in those without sarcopenia (*P = *.038) (Table 2).

**Figure 3:**
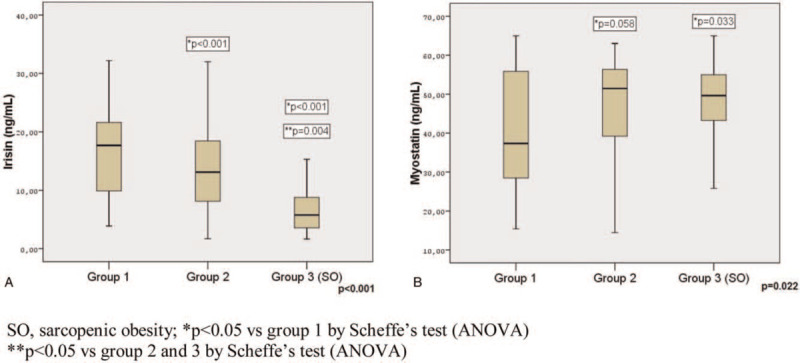
Irisin (A) and myostatin (B) levels according to FM/FFM.

In the evaluation of SO, myostatin levels increased from group 1 to group 3 (*P = *.022), but irisin levels decreased (*P* < .001) (Table 3). In post hoc analysis, myostatin levels were higher (*P = *.058 vs *P = *.033) and irisin levels were lower (*P* < .001 vs *P* < .001) in groups 2 and 3 than in group 1. Irisin levels were significantly lower in group 3 than in group 2 (*P = *.004) (Fig. 3A&B).

We detected a positive correlation between FM/FFM and myostatin and a negative correlation between FM/FFM and irisin (r = 0.303, *P = *.004 vs r = −0.491, *P* < .001). There was a negative correlation between myostatin with ASM (%) and HGS (r = −0.351, *P = *.001 vs r = −0.270, *P = *.010). While irisin was positively correlated with ASM (%) and HGS (r = 0.494, *P* < .001 vs r = 0.354, *P = *.001), it was negatively correlated with FM/FFM and GS (r = −0.491, *P* < .001 vs r = 0.275, *P = *.009). There was a negative correlation between myostatin with FFMI and irisin with FMI (r = −0.326, *P = *.002 vs r = −0.428, *P* < .001) (Table 4).

### 3.4 Logistic regression and ROC analysis

In the multiple logistic regression model using the enter method, irisin (OR:1.105; 95% CI:0.965–1.338, *P = *.002) remained a significant predictor of SO after adjusting for confounding variables. In addition, A1c was an independent risk factor for SO development (OR:1.358, *P = *.055) (Table 5).

**Table 5:** Univariate and multiple logistic regression analyses for predicting sarcopenic obesity.

	Univariate analysis			Multivariable analysis		
Variables	*P*	OR	95% C.I.	*P*	OR	95% C.I.
Age (year)	.578	1.015	0.964–1.068			
HGS (kg)	**.020**	0.928	0.871–0.988			
GS (m/s)	**.002**	1.569	1.176–2.092			
Total-C (mg/dL)	.054	1.011	1.000–1.022			
Irisin (ng/mL)	**.000**	1.302	1.038–1.416	**.002**	1.105	0.965–1.338
Myostatin (ng/mL)	.334	0.984	0.953–1.016			
A1c (%)	**.010**	1.378	1.080–1.757	**.055**	1.358	0.993–1.857

All the variables related to sarcopenic obesity were examined (excluding anthropometric measurements) and only those significant at *P* < .05 level are used in univariate analysis. Multiple logistic regression analysis including all the variables in univariate analysis with enter method. *P* < .05 was considered statistically significant. Non-significant variables in multiple logistic regression analysis were not indicated in the table. 95% CI = 95% confidence interval, A1C = glycosylated hemoglobin, B = regression coefficient, GS = gait speed, HGS = hand grip strength, OR = odds ratio, Total-C = total cholesterol.

**Figure 4:**
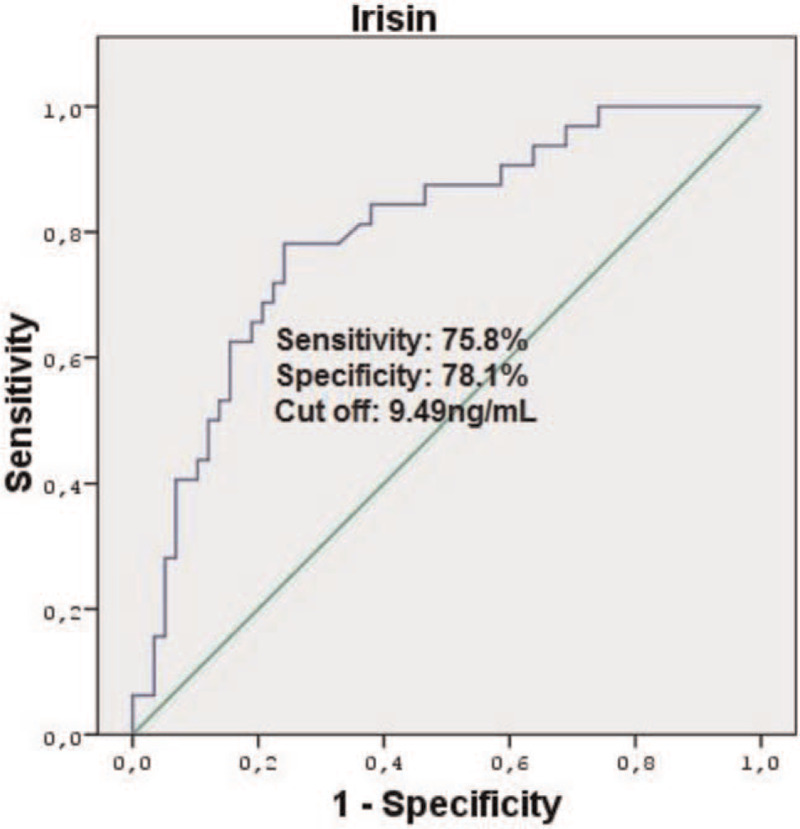
Receiver operator characteristic (ROC) Curve of Irisin to predict SO.

This receiver operating characteristic (ROC) curve shows that the optimal cut-off point of irisin in the prediction of SO was 9.49ng/mL, with a specificity of 78.1% and sensitivity of 75.8% (area under the curve = 0.797; 95% CI:0.703–0.892; *P* < .001) (Fig. 4).

## 4 Discussion

The measurement of body composition is important, especially in obesity, and BIA is a non-invasive method used for this purpose.^[32]^ It is recommended to use weight or height-adjusted ASM (ASM/body weight×100 = ASM (%) or ASM/height [kg/m^2^] = SMI) in the evaluation of low muscle mass in obese patients.^[7]^ Several studies have suggested the FM/FFM ratio in defining the SO.^[22,23]^ Since the patients included in our study had a BMI value of 25–40 kg/m^2^, we demonstrated muscle mass loss with ASM (%) measured by BIA. We also determined low muscle strength with a low HGS and/or increased GS. We also defined SO using the FM/FFM ratio obtained using the BIA.

The prevalence of sarcopenia increases with age.^[33]^ Several studies have revealed an association between diabetes and sarcopenia, and the prevalence of sarcopenia increased 2–3 times in patients with diabetes than in healthy controls.^[3,34]^ However, information on the prevalence of SO in patients with T2DM is limited. In a study that included 198 patients with diabetes, the prevalence of sarcopenia was 29.3%.^[35]^ In another study involving diabetic patients, the prevalence of sarcopenia was found to be 28.8%, 35.6%, and 23.3% in men and women, respectively.^[36]^ In the Korean Sarcopenic Obesity Study, performed by Kim et al^[6]^ the prevalence of sarcopenia was higher in patients with T2DM than in the control group (15.7% vs 6.9%). In another study, when evaluated by sex, the prevalence of SO was 16.7% in men and 5.7% in women with SMI defined sarcopenia; however, it was 35.1% in men and 48.1% in women by ASM (%) defined sarcopenia.^[5]^ Low et al^[37]^ reported the prevalence of SO defined by using the FM/FFM ratio as 19.4% in their study with 1235 T2DM patients. In our study, we found the total prevalence of sarcopenia to be 25.6%, while the SO prevalence was 35.6%. In terms of sex, the prevalence of sarcopenia was higher in females, but the difference was not statistically significant (28.6% vs 15.0%). The prevalence of SO was significantly higher in females than males (41.4% vs 15.0%). In terms of muscle function indicators, the rate of women with low HGS was higher than that of men (54.3% vs 25.0%). Our findings supported that sarcopenia and especially SO prevalence was higher in female patients with T2DM, similar to the literature. We think that the reason for the differences in the prevalence of sarcopenia and SO in the studies is due to the heterogeneity of the diagnostic methods, definitions, and study populations used in the studies.

T2DM and obesity may have harmful effects on muscle mass and function.^[5]^ In contrast, in sarcopenia, skeletal muscle mass, which plays an important role in glucose metabolism, decreases, thereby increasing the risk of IR, obesity, and metabolic syndrome.^[6]^ In our study, BMI, WC, CC, FM, and FMI were significantly higher in patients with sarcopenia than in those without sarcopenia. In addition, we found a negative relationship between FMI, BMI and muscle mass, muscle strength, and a positive relationship between GS. Similar to the literature, our findings support that obesity negatively affects muscle mass and strength in type 2 diabetic patients.

Myostatin is a negative regulator of muscle mass, as well as a potential biomarker that contributes to both metabolic and anabolic defects in SO.^[8,9]^ Some studies have revealed an association between decreased myostatin levels and age, decreased muscle mass, and decreased muscle strength.^[9,10]^ Moreover, high myostatin levels have been shown to be associated with obesity and IR. It was observed that the circulating myostatin concentrations correlated with IR, myostatin expression and secretion increased in skeletal muscle and adipose tissue samples obtained from obese and severely obese women.^[11]^ Myostatin levels are increased in T2DM patients.^[38]^ In our study, myostatin levels were similar in patients with and without sarcopenia, which may be due to the low number of cases in our study. On the other hand, myostatin levels were significantly higher SO (group 3) group than in the control group (group 1). There was a positive correlation between myostatin and FM/FFM and a negative correlation between myostatin and ASM (%), FFMI and HGS. Our data support that myostatin may be an influencing factor in SO.

Irisin is a myokine associated with increased energy expenditure due to its ability to stimulate browning of white adipose tissue, which is secreted after exercise.^[39]^ Irisin levels are reported to be low in obese individuals, patients with T2DM, and coronary artery disease.^[40–42]^ Liu et al^[41]^ showed that irisin levels were lower in long-standing T2DM patients than in non-diabetic controls. Another study found that circulating irisin levels were negatively correlated with BMI, WHR, and FM.^[42]^ Furthermore, a positive correlation has been reported between irisin levels and muscle mass and strength in humans and a negative correlation between irisin levels and fasting blood glucose levels.^[43]^ In our study, irisin levels were significantly lower in patients with sarcopenia. Irisin levels were significantly lower in SO group (group 3) than in the control and group 2. Additionally, we detected a positive correlation between irisin and muscle mass (ASM%) and muscle strength (HGS), while irisin were negatively correlated with poor physical performance (GS), FM/FFM and FMI. In our study, the level of irisin in the development of sarcopenia was an independent risk factor, even after correcting for diabetes-related clinical and laboratory confounding factors. We did not find a cut-off value for irisin for predicting diabetic SO in the literature. In our study, we found that a cut-off value of  < 9.49ng/mL may be a predictor for SO. Our findings suggest that irisin is an important marker for both sarcopenia and SO development in patients with T2DM.

Hyperglycemia also contributes to the development of sarcopenia.^[44,45]^ Several studies have revealed an association between poor glycemic control and sarcopenia. Park et al^[18]^ showed that poor glycemic control (A1c>8.0%) was associated with poorer muscle quality. Sugimato et al^[46]^ showed that there was a positive association between sarcopenia and A1c levels, and poor glycemic control was associated with low muscle mass. In our study, A1c levels were significantly higher in sarcopenic patients than in patients without sarcopenia, and A1c levels in 91.3% of sarcopenic patients were not targeted. Furthermore, there was a positive association between A1c, FM/FFM, and GS, and a negative association between A1c and muscle mass (ASM%), FFMI and muscle strength (HGS). A1c levels were significantly higher in the SO group than in the control group. In addition, A1c was an independent risk factor for SO development (OR:1.358, *P = *.055). Therefore, the relationship between sarcopenia and SO and poor glycemic control may also be an indication that sarcopenia may be reversible after glycemic control is achieved. In a 6-month study, after glycemic control was achieved, a significant increase in muscle mass was observed.^[47]^

As a result, in this study, we showed that myostatin and irisin are effective in sarcopenic and SO patients with type 2 diabetes. We determined that low irisin levels were an independent risk factor, especially for SO, and poor glycemic control (A1c≥7%) had a negative effect on sarcopenia. Therefore, we believe that irisin can be used in the evaluation of SO in patients with type 2 diabetes and that glycemic control is an effective factor in preventing the development of sarcopenia.

### 4.1 Limitations and prospects

First, the sample size included in this study was relatively small, and we planned to increase the sample size in future studies.

Second, a prospective evaluation of the effect of establishing optimal glycemic control with diabetes treatment on the normalization of sarcopenia, irisin, and myostatin levels can be an important contribution.

Third, both irisin and myostatin can be measured using both ELISA and mass spectrophotometry. In our study, we measured both parameters (myostatin and irisin) using an ELISA device with commercial kits. Our results and standard graphics were fine. In our study, we did not compare the methods used for myostatin and irisin. However, these methods can be compared in future studies.

Fourth, although it is not the gold standard method for measuring body composition, we used BIA, which is an easily applicable noninvasive method. This may be a limitation of our study.

## Author contributions

**Conceptualization:** Ayten Oguz.

**Formal analysis:** Ayten Oguz, Dilek Tuzun.

**Funding acquisition:** Ayten Oguz.

**Investigation:** Ergul Belge Kurutas, Cansu Ulgen, Ozlem Bozkus.

**Methodology:** Ayten Oguz, Dilek Tuzun.

**Project administration:** Ayten Oguz.

**Resources:** Ergul Belge Kurutas, Ozlem Bozkus.

**Supervision:** Kamile Gul.

**Validation:** Ergul Belge Kurutas, Ozlem Bozkus.

**Visualization:** Murat Sahin, Dilek Tuzun.

**Writing – original draft:** Ayten Oguz, Murat Sahin, Kamile Gul.

**Writing – review & editing:** Murat Sahin, Cansu Ulgen, Kamile Gul.
